# Neratinib plus dasatinib is highly synergistic in HER2-positive breast cancer *in vitro* and *in vivo*

**DOI:** 10.1016/j.tranon.2024.102073

**Published:** 2024-08-26

**Authors:** Neil T Conlon, Sandra Roche, Amira F Mahdi, Alacoque Browne, Laura Breen, Johanna Gaubatz, Justine Meiller, Fiona O'Neill, Lorraine O'Driscoll, Mattia Cremona, Bryan T Hennessy, Lisa D Eli, John Crown, Denis M Collins

**Affiliations:** a Life Sciences Institute, Dublin City University, Glasnevin, Dublin, Ireland; b School of Pharmacy and Pharmaceutical Science & Trinity Biomedical Sciences Institute, Trinity College Dublin, Dublin, Ireland; c Molecular Medicine - Laboratory of Molecular Oncology, Royal College of Surgeons in Ireland, Dublin, Ireland; d Puma Biotechnology, Inc., 10880 Wilshire Boulevard, Suite 2150, Los Angeles, CA, 90024, USA; e Department of Medical Oncology, St Vincent's University Hospital, Dublin, Ireland

**Keywords:** Breast cancer, HER2-positive, Targeted therapies, Tyrosine kinase inhibitors, Neratinib, Dasatinib

## Abstract

**Background:**

HER2-targeted therapies have revolutionised the treatment of HER2-positive breast cancer. However, *de novo* resistance or the emergence of acquired resistance is a persistent clinical problem. Here we report that neratinib, an irreversible pan-HER inhibitor, in combination with the multi-kinase inhibitor dasatinib, currently used to treat certain leukemias, has strong anti-proliferative effects against models of HER2-positive breast cancer that are innately resistant to trastuzumab or have acquired resistance to neratinib.

**Methods:**

Neratinib plus dasatinib was examined in a panel of 20 breast cancer cell lines, including HER2-positive, estrogen-receptor-positive, triple negative, and acquired HER2-targeted therapy resistant models. Drug effects on migration and apoptosis induction was evaluated and signaling alterations were determined by reverse phase protein array (RPPA). *In vivo* efficacy was examined using orthotopically-implanted HCC1954 cells.

**Results:**

Synergy was observed in cell lines innately resistant to trastuzumab, models with acquired resistance to neratinib, and in triple negative breast cancer cell lines. Further investigation showed that neratinib plus dasatinib induced apoptosis and inhibited cell migration to a greater degree than either drug alone. RPPA revealed that the combination caused suppression of key survival signaling through EGFR, Akt, and MAPK inhibition. *In vivo*, neratinib plus dasatinib was well tolerated and had a prolonged anti-tumor effect against HCC1954 xenografts.

**Conclusions:**

This study provides a strong pre-clinical rationale for the clinical investigation neratinib and dasatinib in HER2+ breast cancer.

## Highlights

• This study provides a comprehensive pre-clinical assessment of neratinib and dasatinib in models of HER2-positive breast cancer.
• The combination of neratinib plus dasatinib was highly synergistic across breast cancer subtypes, with particular sensitivity observed in innately trastuzumab-resistant and acquired neratinib-resistant HER2-positive breast cancer models.
• Neratinib plus dasatinib prevented the emergence of neratinib resistance *in vitro* and *in vivo*.
• Neratinib plus dasatinib was well tolerated and showed a prolonged anti-tumour growth effect *in vivo*.

## Background

Over-expression of human epidermal growth factor receptor 2 (*HER2/ERBB2*) occurs in approximately 20 % of all breast cancers [1]. Historically, HER2-positive (HER2+) breast cancer was classified as more aggressive and patients with this type of breast cancer had a poorer prognosis [2]. However, the development of HER2-targeted therapies over the past three decades has transformed the natural history of HER2+ disease [3]. Several HER2-targeting modalities have now been employed including monoclonal antibodies (*i.e.* trastuzumab, pertuzumab, margutuzumab), antibody-drug conjugates (*i.e.* trastuzumab emtansine, trastuzumab deruxtecan), and tyrosine kinase inhibitors (TKIs). These TKIs are small molecule inhibitors that bind to the intracellular kinase domain of HER2 and may also target other members of the HER family [4]. There are currently three approved HER2-targeting TKIs for HER2+ breast cancer following progression on HER2-targeted antibody-based treatments [[5], [6], [7]]. Lapatinib was the first HER2-targeting TKI approved for the treatment of HER2+ breast cancer and is a reversible dual inhibitor of epidermal growth factor receptor (EGFR) and HER2 [8]. Tucatinib was approved by the FDA in 2020 in combination with trastuzumab and capecitabine in HER2+ breast cancer [5]. Tucatinib is a selective, reversible HER2 inhibitor, with reduced activity against EGFR [9]. Neratinib is an irreversible pan-HER family TKI that has been approved for the treatment of early-stage HER2+ breast cancer following adjuvant trastuzumab, and in combination with capecitabine for the treatment of metastatic HER2+ breast cancer after at least two prior HER2-targeted therapies [10,11].

Our previous pre-clinical comparison of the three approved HER2-targeting TKIs showed that neratinib was the most effective TKI against HER2+ breast cancer models and displayed greater efficacy across multiple cancer types compared to tucatinib and lapatinib [12]. The NALA (NCT01808573) phase III clinical trial provided clinical evidence that neratinib was more effective than lapatinib when combined with capecitabine in HER2+ breast cancer patients, extending progression-free survival (PFS) (12 month PFS = 29 % *versus* 15 %). Despite this improvement, the median duration of response to neratinib was 8.5 months and 12-month overall survival was 72.5 % [11]. This suggests that new treatment options are still needed for HER2+ breast cancer that is refractory to current therapies.

Compensatory signaling networks can circumvent the anti-proliferative effect of HER2-targeted therapies [13,14]. One key signaling node that has been previously associated with HER2-targeted therapy resistance is the proto-oncogene tyrosine kinase Src. The potential of targeting Src in cancer has been explored for forty years [15]. While there are no clinically approved Src-specific inhibitors, dual Src/c-abl inhibitors have been approved for the treatment of leukemias. Dasatinib is a small molecule dual Src/c-abl inhibitor, which also targets several other kinases including other Src family kinases (Lck, Blk, Yes, etc.), PDFGR, c-Kit, and Ephrin family proteins [16,17]. Dasatinib is FDA-approved for the treatment of BCR-Abl-positive chronic myeloid leukemia and acute lymphoblastic leukemia [18]. Previous pre-clinical studies have highlighted the potential of dasatinib to overcome resistance to the HER2-targeted therapies trastuzumab, lapatinib, and afatinib [[19], [20], [21]]. In addition, dasatinib has been tested clinically in combination with trastuzumab and paclitaxel in patients with metastatic HER2+ breast cancer, displaying efficacy with manageable toxicity [22]. It has also been safely combined with the pan-HER inhibitor afatinib in patients with non-small cell lung cancer [23].

In this pre-clinical study, the efficacy of combining neratinib and dasatinib is investigated in HER2+ breast cancer models that (1) are naïve to therapy, (2) have acquired resistance to trastuzumab, and (3) are acquired neratinib-resistant. This study examines the anti-proliferative effect of this novel drug combination *in vitro* and *in vivo* and explores the ability of neratinib plus dasatinib to induce apoptosis, prevent migration, and suppress key signaling pathways.

## Methods

### Reagents

Neratinib (provided by Puma Biotechnology), lapatinib, dasatinib (Carbosynth), bosutinib, imatinib, pyrotinib, afatinib, and tucatinib (SelleckChem) were prepared as 10 mM stocks in dimethyl sulfoxide (DMSO). Trastuzumab (St. Vincent's University Hospital) was prepared to 21 mg/mL working stock.

### Cell culture

HCC1954, HCC1569, JIMT-1, SKBR3, EFM192A, and BT474 cell lines were obtained from the American Type Culture Collection (ATCC) and cultured in RPMI-1640 supplemented with 10 % foetal bovine serum (FBS) (Gibco). All cell culture and experimental assays were incubated in a humidified atmosphere with 5 % CO_2_ at 37 °C. Generation of the acquired trastuzumab-resistant cell lines SKBR3-T and BT474-T has been described previously [14]. The neratinib-resistant cell lines, HCC1954-N, EFM192A-N, and SKBR3-N, were generated by continuous exposure to neratinib as previously described [24,25]. The HCC1569-N and BT474-N were produced by continuous exposure to escalating doses of neratinib (5–150 nM and 5–80 nM, respectively) over six months.

All cell lines were routinely tested for mycoplasma in-house and authenticated by short tandem repeat profiling (Source BioScience). Drug-resistant cell lines were maintained in their respective drugs until one week before all experiments, when cells were withdrawn from neratinib or trastuzumab treatment.

### Proliferation assay

Cell proliferation was assessed using an acid phosphatase-based proliferation assay. 2.5 – 5 × 10^3^ cells/well were seeded into a 96-well plate and incubated overnight. Combination drug effects were assessed by either 1:1 ratio or by combinatorial matrices. Drugs were added to each plate and incubated for a further five days. Medium was then removed and cells were washed with phosphate buffer saline (PBS). 100 µL para-nitrophenol phosphate substrate (Sigma Aldrich) in 0.1 M sodium acetate buffer with 0.1 % Triton X-100 (Sigma Aldrich) was added to each well and incubated at 37 °C for 60–90 min. 50 µL of 1 M sodium hydroxide was added to each well. The absorbance was read using a spectrophotometer (BioTek Synergy HT) at 450 nm and 620 nm as a background reference. Growth of drug-treated cells was calculated as a percentage relative to untreated control cells in biological triplicate. 1:1 ratio proliferation assays were analyzed using GraphPad Prism and CalcuSyn software. Combenefit software was used to calculate Loewe synergy scores for combinatorial matrix assays [48].

### Short-term neratinib resistance assay

Cells were seeded into two 12-well plates and incubated overnight at 37 °C. Medium was replenished (control) or treated with 150 nM neratinib, 150 nM dasatinib or the combination. Plates were treated twice weekly. Plate 1 was fixed with 4 % para-formaldehyde and stained with 0.1 % crystal violet when untreated control cells reached confluence. Plate 2 continued treatment until single agent neratinib resistance emerged. The plate was then fixed and stained with crystal violet. 10 % acetic acid was used to elute the crystal violet and absorbance was read at 570 nm on a spectrophotometer (BioTek Synergy HT).

### Reverse phase protein array

HCC1954 and HCC1954-N cells were seeded into 10 cm petri dishes and incubated at 37 °C overnight. Once cells reached 80 % confluence, they were treated with DMSO, 150 nM neratinib, 150 nM dasatinib, or 150 nM neratinib plus 150 nM dasatinib for 24 h. Cells were then washed with PBS and lysed in RPPA buffer (1 % Triton X-100, 50 mM HEPES pH 7.4, 150 mM NaCl, 1.5 mM MgCl_2_, 1 mM EGTA, 100 mM NaF, 10 mM sodium pyrophosphate tetrabasic, 1 mM sodium orthovanadate, 10 % glycerol) containing protease (cOmplete, Roche Life Science) and phosphatase (phosSTOP, Roche Life Science) inhibitors. Reverse phase protein array (RPPA) on biological quadruplicate lysates was performed as previously described by [26]. Briefly, a sample array was created using a 2470 arrayer (Aushon BioSystem) on Oncocyte Avid nitrocellulose coated slides. Immunostaining was performed on an automated slide stainer (Dako Link 48) according to manufacturer's instructions. Primary antibody incubation was carried out a room temperature for 30 min followed by anti-mouse or anti-rabbit secondary antibody. Images of slides were analyzed using Microvigene software (v5.1). Data was analyzed using GraphPad Prism (v.9).

### Caspase 3/7 analysis

Cell proliferation and apoptosis was measured using the Incucyte S3 live-cell imaging system (Essen Biosciences). 3 × 10^3^ cells/well were seeded into a 96-plate and incubated overnight. Drugs were prepared in medium with Incucyte Caspase 3/7 green dye. Drugs were added to the plate and cell growth and caspase 3/7 activation were monitored every 6 h using the Incucyte. Data were analysed using GraphPad Prism (v.9).

### Scratch wound migration assay

Cells were seeded at 1 × 10^4^ cells/well in a 96-well plate and allowed to reach confluency. Scratches were applied to each well using the Incucyte WoundMaker tool. Cells are washed with PBS to remove floating cells. 200 μL of medium, 150 nM neratinib, 150 nM dasatinib, or neratinib plus dasatinib was added to each well. Scratch closure was monitored every 6 h using the IncuCyte S3 system. GraphPad Prism was used to visualize drug effect on wound confluency.

### *In vivo* assessment of neratinib and dasatinib

All *in vivo* work was carried out in Dublin City University and was approved by DCU Research Ethics Committee (DCUREC/2018/133) and the Health Product Regulatory Authority (AE19115_P026). All mice were housed in groups of up to five in individually ventilated cages in a specific pathogen-free unit. As the *in vitro* experiments examined female breast cancer models, only female mice were used. Bedding material, environmental enrichment, and access to water and food pellets were available in all cages. BALB/c nu/nu mice (Charles River, UK) were implanted with 7.5 × 10^5^ HCC1954 cells into the right inguinal mammary fat pad, using a 25-gauge needle. Animals were randomized to four treatment arms of 5–6 mice (vehicle, 10 mg kg^-1^ neratinib, 15 mg kg^-1^ dasatinib, neratinib plus dasatinib) when average tumor volume reached 200 mm^3^. Neratinib, dasatinib or vehicle (0.5 % methocellulose-0.4 % Tween-80 in water) was administered by oral gavage on a 5-days on, 2-days off schedule. The total volume given per gavage was 200 µL. Tumour growth and weight changes were monitored three times per week by a treatment-arm-blinded researcher and tumor volume was calculated as (Width x Height x Depth)/1.9. Animals were euthanized by cervical dislocation when any of the humane endpoints were reached. Humane endpoints were defined as: tumor volume greater than 1600 mm^3^, a tumor dimension exceeding 15 mm, a decline in general health, loss of body weight exceeding 20 % of weight at start of treatment, or loss of skin integrity. Statistical significance was tested by two-way ANOVA.

Tumors were retrieved for *ex vivo* analysis by enzymatic digestion in a hyaluronidase:collagenase mixture (Sigma). After 1 h incubation at 37 °C, cells undergoing enzymatic digestion were centrifuged at 1000 rpm for 5 min, resuspended in RPMI-1640 medium supplemented with 10 % FBS. Cells were allowed to attach overnight at 37 °C. Tumours were also fresh frozen in liquid nitrogen for Western blotting analysis. Fresh frozen samples were powdered in liquid nitrogen using a pestle and mortar. RIPA lysis buffer supplemented with protease inhibitor cocktail (Sigma), sodium orthovanadate, and PMSF was added to powdered tumors. Samples were mixed, centrifuged at 4 °C and protein quantified by BCA assay (ThermoFisher). Equal amounts of protein sample were prepared in 4X SDS buffer (Invitrogen), denatured, and loaded into a Bolt 4–12 % gel. Proteins were separated by SDS-PAGE gel electrophoresis and transferred using the iBlot transfer system (Invitrogen). EGFR, pEGFR, HER2, pHER2, Src, pSrc, and alpha tubulin primary antibodies and mouse and rabbit secondary antibodies (LiCor) were used. Blots were imaged on a LiCor Odyssey.

## Results

### Dasatinib enhances neratinib response in HER2-positive breast cancer cell lines with moderate neratinib sensitivity

Sensitivity to neratinib, dasatinib, and the combination was first assessed in 16 breast cancer cell lines representing the three main clinical subtypes: eight HER2+ breast cancer cell lines (including models of innate and acquired trastuzumab-resistance), six triple negative breast cancer (TNBC) cell lines, and two estrogen receptor-positive (ER+) breast cancer models (Table 1). The HER2+ cell line panel included cell lines that are known to be highly sensitive to HER2-targeted therapies (BT474, EFM192A, and SKBR3), innately resistant to trastuzumab (HCC1954, HCC1569, and JIMT-1), and models of acquired trastuzumab resistance (BT474-T and SKBR3-T) . As expected, neratinib alone was highly potent against all HER2+ breast cancer cell lines (IC_50_ value range = 1.2 – 170 nM neratinib). Neratinib IC_50_ values for acquired trastuzumab resistance models were similarly in the nanomolar range - BT474-T (IC_50_ value=5.4 ± 1.5 nM) or SKBR3-T (IC_50_ value= 4.2 ± 3.8 nM). This is in agreement with previous pre-clinical and clinical results [14,27]. As expected, neratinib displayed higher IC_50_ values (> 1 µM) in both HER2-ER+ cell lines tested (MCF7 and ZR-75–1). Sensitivity to neratinib in TNBC cell lines was similar to that found in previous testing with afatinib [28]. Neratinib IC_50_ values in the TNBC cell lines ranged from 31.1 nM to 629.7 nM (Table 1).

**Table 1:** Sensitivity to neratinib, dasatinib, and the combination in the panel of HER2+ (including acquired (BT474-T, SKBR3-T) and innately (JIMT-1, HCC1954, HCC1569) trastuzumab-resistant models), estrogen receptor-positive (ER+) and triple negative (TN) breast cancer cell lines. All results are independent triplicate experiments ± standard deviation.

**Breast cancer subtype**	**Cell line**	**Neratinib IC_50_ values (nM)**	**Dasatinib IC_50_ values (nM)**	**CI value**
HER2+	BT474	1.2 ± 0.3	>150	N/A
	EFM192A	2.2 ± 0.8	>150	N/A
	SKBR3	12.3 ± 2.2	>150	N/A
	BT474-T	5.4 ± 1.5	>150	N/A
	SKBR3-T	4.2 ± 3.8	>150	N/A
	JIMT-1	172.0 ± 78.2	112.9 ± 42.7	0.42 ± 0.09
	HCC1954	9.4 ± 0.5	318.0 ± 149.3	0.62 ± 0.11
	HCC1569	26.2 ± 5.7	>1000	0.27 ± 0.02
ER+	MCF7	>1000	337.2 ± 165.9	0.29 ± 0.08
	ZR-75–1	>1000	>1000	N/A
TN	BT20	629.7 ± 120.0	>1000	0.17 ± 0.03
	HCC1143	379.4 ± 106.5	122.2 ± 8.9	0.66 ± 0.17
	HCC1937	226.6 ± 72.0	135.6 ± 56.0	0.36 ± 0.06
	MDA-MB-157	>1000	9.0 ± 1.9	N/A
	MDA-MB-231	>150	8.6 ± 0.9	N/A
	MDA-MB-468	31.1 ± 6.9	>1000	1.18 ± 0.23

Dasatinib had little effect as a single agent across the breast cancer cell line panel. An IC_50_ value was not determinable for 6/8 HER2+ cell lines as dasatinib did not achieve 50 % growth inhibition at the concentrations tested. TNBC cell lines were the most responsive to dasatinib (IC_50_ values ranging from 8.6 nM to >1 µM), with the mesenchymal stem–like cell lines MDA-MB-157 and MDA-MB-231 displaying low nanomolar sensitivity. This has been previously observed in TNBC pre-clinical testing [29].

**Fig. 1:**
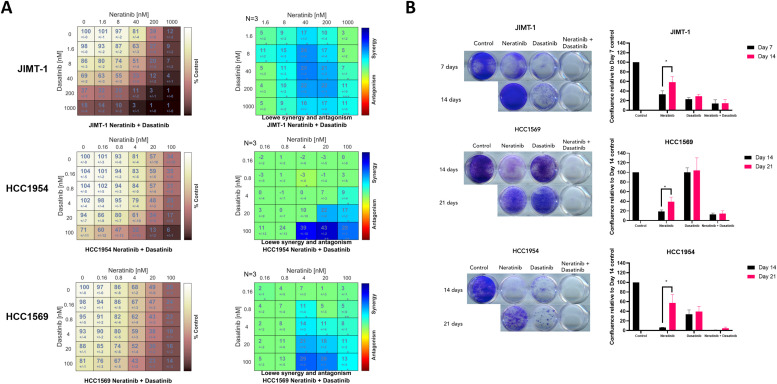
Neratinib plus dasatinib is highly synergistic in innately trastuzumab-resistant cell lines. (A) Concentration-dependent synergy between neratinib and dasatinib was examined by matrix assays in JIMT-1, HCC1954, and HCC1569 cell lines. (B) The addition of dasatinib delays the onset of neratinib resistance. Cells were treated twice weekly. Plate 1 was fixed and stained with crystal violet when control cells reached confluence. Plate 2 was stained when cells no longer responded to neratinib alone. Standard deviation error bars are representative of independent triplicate experiments. Mann Whitney U test, *p* < 0.05 is significant.

Combination index (CI) values are only determinable for combinations where both drugs have growth inhibitory effects. The synergistic activity of neratinib plus dasatinib across the breast cancer panel is reported in (CI/IC_50_ values in Table 1, graphs in Supplemental figures 1 and 2). Strong synergy was observed in the HER2+ cell lines that are innately resistant to trastuzumab: HCC1569, HCC1954, and JIMT-1 (Table 1 - CI values ranging 0.27–0.62). This synergistic interaction was further assessed by matrix proliferation assays (Fig. 1A). This analysis revealed that neratinib and dasatinib display synergy at low nanomolar concentrations, and no antagonism is observed with any ratio of the two drugs examined. Synergy was most notably observed with concentrations of 4–100 nM dasatinib in HCC1954 and HCC1569 cells. Cell lines that displayed potent sensitivity to either single agent did not show synergism. The high potency of single agent neratinib in BT474, EFM192A, SKBR3, BT474-T, and SKBR3-T cells provided no window for additional benefit from dasatinib in these cell lines. As dasatinib did not have a growth inhibitory effect on these five cell lines, CI values could not be calculated.

One of the two HER2-ER+ cell lines, MCF7, displayed a synergistic response to the combination; however, this was at concentrations higher than clinically achievable. Synergy was also observed in 3/6 TNBC cell lines (Table 1 and Supplementary Figure 2). The other three TNBC cell lines were sensitive to either single agent neratinib or dasatinib treatment at low nanomolar concentrations (8.6 – 31.1 nM). This was similar to what we have previously seen with afatinib plus dasatinib [30].

### Neratinib and dasatinib displayed synergy and prolonged anti-proliferative effect in innately trastuzumab-resistant cell lines at nanomolar concentrations

Cancer cell lines can develop resistance to targeted therapies with continued exposure to the drugs. In order to model this phenomenon *in vitro*, two 12-well plates of HCC1549, HCC1954, and JIMT-1 cells were treated with 150 nM neratinib, 150 nM dasatinib, or the combination twice weekly (Fig. 1B). Treatment of the first plate was ended when untreated cells reached confluency. All three cell lines showed sensitivity to neratinib and the combination. The second plate continued twice-weekly treatment for a further week (JIMT-1) or two weeks (HCC1569 and HCC1954) when neratinib resistance began to emerge. All three cell lines treated with neratinib showed increased proliferation compared to the previous timepoint (*p* = 0.03, 0.02, and 0.04, respectively). After the additional treatments, the cell lines had stopped responding to single agent neratinib but the neratinib/dasatinib anti-proliferative effect was sustained.

### Synergy was strongest in combinations of dasatinib and irreversible HER2 TKIs

**Fig. 2:**
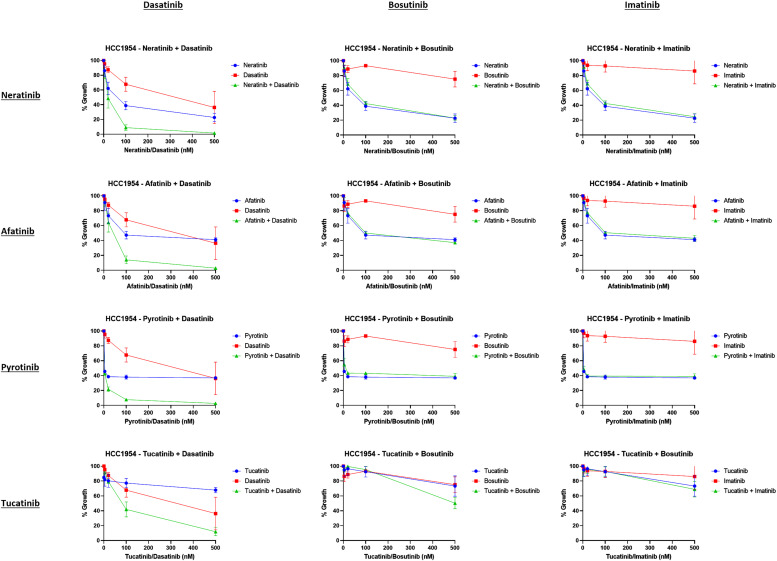
Dasatinib causes greater synergy with HER2 targeted TKIs than bosutinib or imatinib. The anti-proliferative effect of HER2-targeted TKIs neratinib, afatinib, pyrotinib, and tucatinib, and the multi-kinase inhibitors dasatinib, bosutinib, and imatinib were examined in the HCC1954 cell line. Synergy was determined by fixed ratio CI values. All results are based on independent triplicate experiments ± standard deviation.

As previously discussed, there are no clinically approved Src-specific inhibitors. To determine if the synergy observed in HER2+ breast cancer models is specific to neratinib and dasatinib, we wished to evaluate the effectiveness of (1) different HER2-targeted TKIs in combination with dasatinib and (2) other Src/c-abl inhibitors in combination with HER2-targeted TKIs. Combinations involving the HER-family targeting TKIs afatinib (pan-HER, irreversible TKI), pyrotinib (pan-HER, irreversible TKI) and tucatinib (HER2-specific, reversible TKI) and two other Src/c-abl inhibitors, bosutinib and imatinib (Fig. 2) were examined in the HCC1954 cell line. Similar to neratinib, HCC1954 cells were sensitive to afatinib (IC_50_ value = 159.3 ± 53.7 nM) and pyrotinib (IC_50_ value <4 nM). HCC1954 cells were more resistant to tucatinib achieving 26.7 ± 11.2 % growth inhibition at the maximum tested concentration (500 nM). Synergy was observed between dasatinib and all HER2-targeted TKIs tested (Fig. 2). In contrast, bosutinib and imatinib had minimal activity as single agents (IC_50_ values > 500 nM) and did not enhance the efficacy of the HER2-targeted TKIs tested, including neratinib.

Anti-proliferative synergy was also examined in combination with other Src inhibitors in HCC1954 and HCC1569 cells. These included CCT196969 (pan-Raf/Src TKI), SU6656 (pan-Src family), and TPX-0005 (ROS1/TRK/ALK/Src inhibitor) (Supplementary Figure 3). These drugs did not achieve synergy in combination with neratinib. Therefore, the combination of neratinib and dasatinib was progressed for further characterization.

### The addition of dasatinib overcomes acquired neratinib resistance

**Fig. 3:**
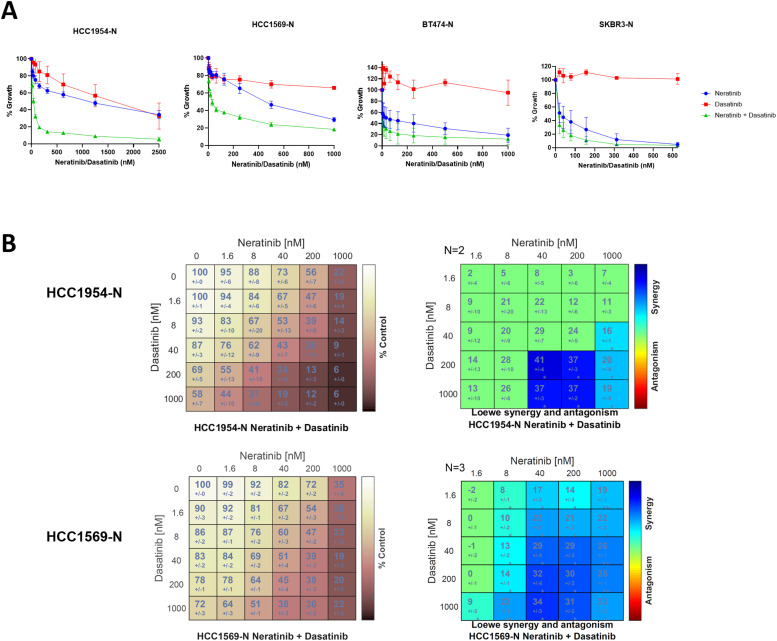
Neratinib plus dasatinib is highly synergistic in neratinib-resistant breast cancer cell lines. The anti-proliferative effect of neratinib and dasatinib was examined in (A) four cell lines with acquired neratinib resistance. Error baes are representative of independent triplicate experiments ± standard deviation. (B) Concentration-dependent synergy was examined by matrix assays in HCC1954-N and HCC1569-N cell lines. Results consist of independent triplicate experiments.

Acquired therapy resistance is an important clinical challenge and a major reason why drugs fail in the clinic. Data in Fig. 1B shows that neratinib/dasatinib can delay the onset of neratinib resistance in models of innate resistance. We aimed to determine if dasatinib could overcome, as well as prevent, neratinib resistance. A panel of four acquired neratinib-resistant HER2+ breast cancer cell lines (HCC1954-N, HCC1569-N, BT474-N, SKBR3-N) was investigated for the potential for the neratinib/dasatinib combination in models of neratinib-refractory disease. All cell lines tested are resistant to neratinib at clinically relevant concentrations (IC_50_ values >150 nM). The panel includes three HER2+ ER- and one HER2+ ER+ (BT474-N) cell lines. Dasatinib alone resulted in IC_50_ values > 1 µM in 2/4 cell lines, and failed to reach an IC_50_ in three of the cell lines tested (Fig. 3A). However, the combination of neratinib and dasatinib was highly synergistic across all neratinib-resistant cell lines (Fig. 3A). Matrix drug analysis revealed that the combination displayed synergy down to low nanomolar concentrations of neratinib and dasatinib (Fig. 3B). This highlights the ability of neratinib plus dasatinib to overcome neratinib resistance at clinically achievable concentrations.

### The combination of neratinib and dasatinib induced apoptotic cell death

**Fig. 4:**
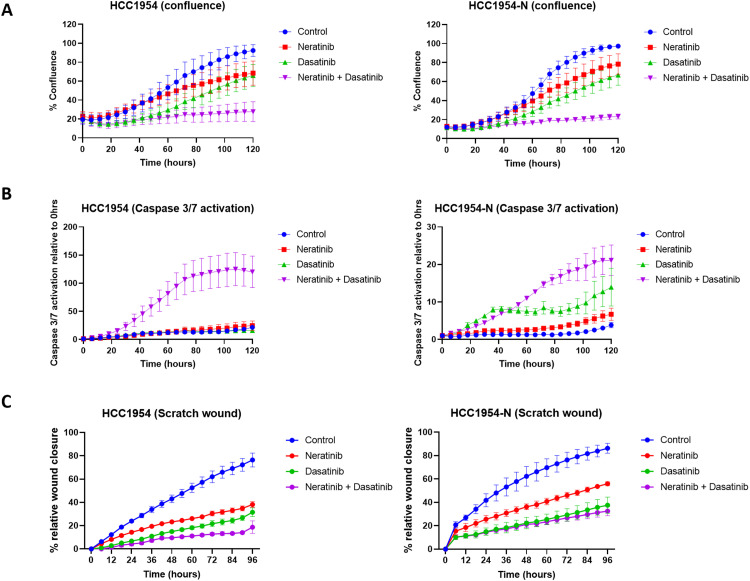
The combination of neratinib and dasatinib results in greater growth inhibition, apoptosis induction, and inhibition of migration compared to single agents. (A) neratinib, dasatinib, and combination effect on cell confluence in HCC1954 and HCC1954-N cells. (B) Caspase 3/7 activation related to apoptosis, relative to cell confluence, in HCC1854 and HCC1954-N cells treated with neratinib, dasatinib, and the combination. (C) Anti-migration effects of neratinib, dasatinib, and combination as measured by scratch wound assay in HCC1954 and HCC1954-N cells. 150 nM neratinib and 150 nM dasatinib were used for all experiments. Error bars are representative of standard error mean independent triplicate experiments.

We wished to examine whether the combination of neratinib and dasatinib induced a cytotoxic or a cytostatic effect on HER2+ breast cancer models. HCC1954 and HCC1954-N cells were selected for further characterization as these cell lines exhibit strong synergy and represent treatment-naïve and neratinib-resistant phenotypes. The HCC1954 and HCC1954-N cells were treated with 150 nM neratinib, dasatinib, or the combination, and the induction of caspase 3/7 activation, an apoptosis marker, was quantified every six hours using the IncuCyte S3 system (Fig. 4A and B). Neratinib plus dasatinib caused significant inhibition of cell growth within 48 h of treatment and this effect was sustained for 120 h in both cell lines (Fig. 4A) (Supplementary Figure 4). Apoptosis was induced in the HCC1954 and HCC1954-N cells within 72 h of treatment and was sustained for the remainder of the assay (Fig. 4B).

The impact of neratinib/dasatinib on cell migration was examined using a scratch wound assay [31]. Neratinib or dasatinib alone had a strong anti-migratory effect on both cell lines (Fig. 4C) (Supplementary Figure 5). Neratinib plus dasatinib enhanced this effect in the HCC1954 cell line. HCC1954-N cells have a higher basal migration level than their parental cell line and neratinib alone has less of an anti-migratory effect on the resistant variant (Supplementary figure 6). However, both dasatinib alone, and neratinib plus dasatinib, caused a three-fold reduction in HCC1954-N cell migration (Fig. 4C).

### RPPA analysis of intracellular signaling impact of neratinib plus dasatinib

**Fig. 5:**
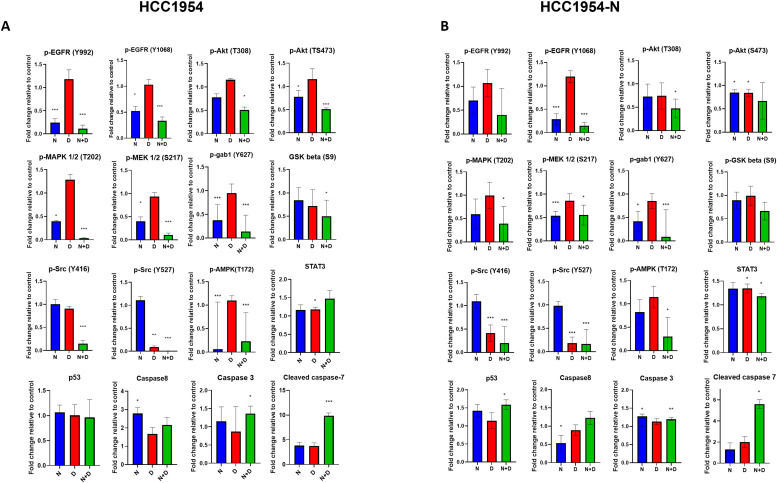
RPPA analysis of neratinib/dasatinib treatment effects reveals enhanced EGFR, Src, and PI3K/MAPK signalling inhibition in (A) HCC1954 and (B) HCC1954-N. Fold change is relative to control cells. Error bars represent standard deviation of independent quadruplicate experiments. Student's t-test, *p < 0.05, **p < 0.01, ***p < 0.001.

In order to investigate the effect of neratinib and dasatinib on intracellular cell signaling, RPPA analysis was performed on HCC1954 and HCC1954-N cells treated with neratinib, dasatinib or the drug combination for 24 h. Neratinib significantly inhibited EGFR phosphorylation (Y992 and Y1068) in HCC1954 cells (Fig. 5A). In contrast, HCC1954-N cells showed a numerical but statistically insignificant reduction in EGFR (Y992) phosphorylation but significant inhibition of EGFR Y1068 phosphorylation (Fig. 5B). The phosphorylation of Akt (T308 and T473), MAPK (T102), MEK 1/2 (S217), and Gab1 (Y627) were examined as markers of activation of the PI3K and MAPK pathways, which are activated by HER family signaling. In both HCC1954 and HCC1954-N cells, greater suppression of PI3K and MAPK signaling was observed with the combination treatment. Furthermore, the addition of neratinib to dasatinib caused a greater degree of inhibition of Src phosphorylation in HCC1954 cells, indicating the combination is enhancing on-target kinase inhibition. Activation of AMPK is reported to decrease EGFR and HER2 activity [32]. However, in this study although AMPK inhibition was observed with neratinib/combination treatment, this did not affect suppression of EGFR activity. STAT3 has been implicated in resistance to lapatinib in HER2+ breast cancer [33]. Total STAT3 levels were significantly decreased with combination treatment in the neratinib-resistant cells. The RPPA analysis also highlighted the apoptosis induction caused by neratinib plus dasatinib as cleaved caspase-7 was elevated following 24 h treatment.

### Neratinib plus dasatinib *in vivo*

**Fig. 6:**
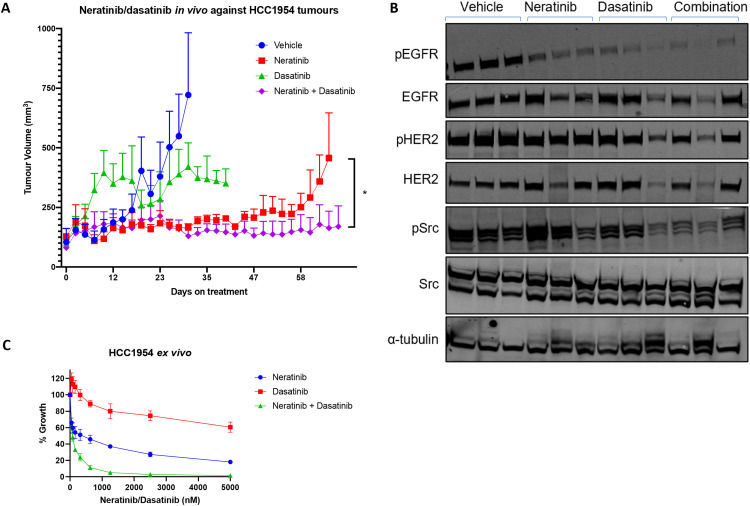
Neratinib plus dasatinib inhibits tumour xenograft growth.(A) HCC1954 cells were implanted into the mammary fat pad of BALB/c nude mice. Mice were divided into four treatment arms: vehicle control, neratinib, dasatinib, and neratinib plus dasatinib. Tumour volume was monitored by caliper measurements. Data was plotted as meant tumour volume ± standard error of the mean. (B) Western blotting analysis of neratinib and dasatinib targets in JCC1954 tumours treated with vehicle, neratinib, dasatinib, or combination. (C) HCC1954 tumours were resected and grown *ex vivo* after 12 weeks of growth *in vivo*. Maintenance of sensitivity was confirmed by 5-day acid-phophastase assay.

The *in vivo* activity of neratinib and dasatinib was investigated in an HCC1954 xenograft model. The HCC1954 cells were implanted into the mammary fat pad of BALB/c nude mice to better mimic the natural microenvironment. Neratinib (10 mg kg^-1^) plus dasatinib (15 mg kg^-1^) was well tolerated for 10 weeks of treatment. The vehicle treated tumors and dasatinib alone arms reached humane endpoints at day 30 and day 40, respectively. Neratinib alone prolonged survival (68 days) compared to vehicle control. Neratinib plus dasatinib had an enhanced anti-tumor effect compared to either single agents (*p* = 0.02) (Fig. 6A). Tumors were resected post-mortem. Tumor material was fresh frozen for protein analysis and HCC1954 cells were dissociated and grown *ex vivo*. Western blotting analysis was performed on resected tumors. This analysis showed decreased EGFR phosphorylation with neratinib alone, dasatinib alone, and combination treatment (Fig. 6B). Combinatorial treatment resulted in statistically significant reduction in HER2 activation and numerically decreased total HER2, EGFR, phosphorylated ERK, and phosphorylated Src compared to single agent treatment (Supplementary Figure 7). The *ex vivo* cells displayed similar morphology to HCC1954 cells not implanted in mice and maintained comparable response to neratinib, dasatinib, and the combination therapy *in vitro* (Fig. 6C). Overall, *in vivo* investigation of neratinib plus dasatinib reiterated the efficacy observed *in vitro*.

## Discussion

This pre-clinical study showed that neratinib and dasatinib have remarkable nanomolar synergistic anti-proliferative activity in models of HER2+ and triple negative breast cancers, and the combination treatment was capable of overcoming HER2-targeted TKI resistance. Both neratinib and dasatinib are approved anti-cancer drugs and this study highlights the potential of repurposing dasatinib as an enhancing agent in the treatment of HER2+ breast cancer.

We previously reported that neratinib has a greater anti-proliferative effect than the other FDA-approved HER2-targeting TKIs lapatinib or tucatinib in a large-scale *in vitro* study. We believe that the irreversible nature of drug-receptor binding and pan-HER family inhibition of neratinib are the underlying mechanisms [4,12]. In this study, the irreversible inhibitors (neratinib, afatinib, pyrotinib) again displayed greater anti-proliferative activity compared to tucatinib (Fig. 2), validating our focus on the pan-HER TKI neratinib.

The *in vitro* anti-proliferative assessment found that all HER2+ cell lines investigated had sub-micromolar sensitivity to neratinib, which included models of acquired and innate resistance to trastuzumab (Table 1). The innately trastuzumab-resistant models (HCC1954, HCC1569, JIMT-1) displayed the greatest synergy when neratinib and dasatinib were combined (Fig. 1) and strong synergy was also observed in the acquired neratinib-resistant cell lines (Fig. 3). Importantly, the combination of neratinib and dasatinib did not result in antagonism at any concentrations examined.

There is extensive evidence of crosstalk between the Src family kinases and HER family members, and Src kinase can act as a compensatory survival signal, as previously reported in resistance mechanisms to trastuzumab, trastuzumab emtansine, lapatinib, afatinib, and neratinib [19,[34], [35], [36]]. Src family kinase Yes1 has been associated with poor survival in HER2+ breast cancer and amplification or mutation can cause neratinib resistance. Inhibition of Yes1, with siRNA or dasatinib, has been shown to overcome this resistance [35]. Our results, further the argument that combinatorial inhibition of the HER family and Src has potential against the HER2+ drug-resistant phenotype, and may also have value delaying the emergence of neratinib resistance (Fig. 1).

It should be noted that the *in vitro* screen also reported synergy for neratinib plus dasatinib in 3/6 of the TNBC cell lines tested, with the remaining cell lines displaying nanomolar sensitivity to single agent treatment (Table 1). Dasatinib has been shown to increase the *in vitro* effectiveness of a broad range of other targeted therapies such as olaparib, trametinib, and selinexor [[37], [38], [39]].We have previously shown that afatinib plus dasatinib is synergistic in TNBC models [40]. EGFR over-expression, correlation between EGFR-HER3 axis activation and poor outcome, and higher levels of cytoplasmic Src in TNBC compared to other breast cancer subtypes also point to potential therapeutic value for a pan-HER inhibitor and Src inhibitor in TNBC [[41], [42], [43]].

We compared the enhancing ability of dasatinib to similar Src–targeting multi-kinase inhibitors bosutinib and imatinib without observing the same synergy in combination with pan-HER TKIs (Fig. 2). Bosutinib and imatinib have distinct kinome inhibitory profiles to dasatinib and are less potent inhibitors of the Src family kinases Src, Lck, and Yes, which may explain the differential effects (Supplementary figure 8). Additional examination of three further compounds with reduced multi-kinase profiles and more Src specificity (CCT196969, SU6656, TPX-0005) once again supports the multi-kinase nature of dasatinib as an important component of its synergistic activity in combination with pan-HER TKIs (Supplementary figure 3).Further investigation of dasatinib targets outside Src family / c-Abl in this context are underway.

Mechanistic studies were undertaken to understand the basis for neratinib/dasatinib synergy. Neratinib plus dasatinib stimulates a strong induction of apoptosis in models of innate trastuzumab-resistance and acquired resistance to neratinib (Fig. 4). The enhanced suppression of cell migration associated with the neratinib/dasatinib combination (Fig. 4) is most likely due to disruption of the HER2, EGFR, and Src interplay associated with cell migration [36,37]. The RPPA data display enhanced inhibition of Src family signaling and augmented suppression of the Akt and MAPK pathways in the presence of the neratinib/dasatinib combination, over either agent alone (Fig. 5). We therefore believe that the basis of neratinib/dasatinib synergy is potent, irreversible pan-HER inhibition combined with Src family/multi-kinase inhibition resulting in enhanced blockade of key survival pathways and the elimination of compensatory resistance signaling pathways (Supplementary figure 9).

The *in vivo* assessment of neratinib plus dasatinib confirmed the efficacy of single agent neratinib and the neratinib/dasatinib combination in a HER2+ breast cancer xenograft model. The dosages of neratinib (10 mg kg^-1^) and dasatinib (15 mg kg^-1^) are relatively low compared to published *in vivo* studies; neratinib has been assessed up to 40 mg kg^-1^ and dasatinib up to 50 mg kg^-1^ *in vivo* [44,45]. However, the concentrations chosen for this study still elicit on-target inhibition of HER family and Src activation in addition to tumor growth suppression (Fig. 6), while being more clinically relevant. Human equivalent dose calculations show that the *in vivo* dosages used in this study are reflective of clinically achievable concentrations - 10 mg kg^-1^ neratinib: 15 mg kg^-1^ dasatinib equivalent to 0.81 mg kg^-1^ neratinib: 1.2 mg kg^-1^ dasatinib in humans [46].

Clinical investigation of HER2-inhibitor/dasatinib combinations have shown them to be safe. The addition of dasatinib to trastuzumab and paclitaxel as a first line treatment of metastatic HER2+ breast cancer was well tolerated, with no grade 4 toxicities, and showed clinical activity (ORR=89 %) [47]. The combination of dasatinib and the irreversible pan-HER TKI afatinib has been examined in a phase I clinical trial (NCT01999985) in a cohort of non-small cell lung cancer patients who had received at least two EGFR-targeting regimens, reporting that a pan-HER inhibitor and dasatinib was well-tolerated.

In summary, this pre-clinical study showed that the approved anti-cancer drugs neratinib and dasatinib are highly synergistic in models of hard-to-treat HER2+ breast cancer. No antagonism was observed across 20 breast cancer cell line models from HER2+, TN, or ER+ subtypes. Neratinib and dasatinib enhanced on-target inhibition, which suppressed key survival signaling and induced apoptotic death. The combination was well-tolerated *in vivo* and had a prolonged anti-tumor effect against a HER2+ breast cancer xenograft. This pre-clinical study suggests that the combination of neratinib and dasatinib is a highly promising novel therapeutic strategy for HER2+ breast cancer, particularly tumors that are innately resistant or have acquired resistance to current HER2-targeted therapies.

## CRediT authorship contribution statement

**Neil T Conlon:** Writing – original draft, Visualization, Project administration, Methodology, Investigation, Formal analysis, Conceptualization. **Sandra Roche:** Writing – review & editing, Methodology, Investigation. **Amira F Mahdi:** Writing – review & editing, Investigation. **Alacoque Browne:** Writing – review & editing, Investigation. **Laura Breen:** Writing – review & editing, Investigation. **Johanna Gaubatz:** Writing – review & editing, Investigation. **Justine Meiller:** Writing – review & editing, Investigation. **Fiona O'Neill:** Writing – review & editing, Investigation. **Lorraine O'Driscoll:** Writing – review & editing, Investigation. **Mattia Cremona:** Writing – review & editing, Investigation. **Bryan T Hennessy:** Writing – review & editing, Investigation. **Lisa D Eli:** Writing – review & editing, Resources, Project administration. **John Crown:** Writing – review & editing, Supervision, Funding acquisition, Conceptualization. **Denis M Collins:** Writing – original draft, Supervision, Project administration, Funding acquisition, Conceptualization.

## Declaration of competing interest

The authors declare the following financial interests/personal relationships which may be considered as potential competing interests: The conflict of interest for this study are that DMC, NTC and JC have received academic research funding from Puma Biotechnology Inc., DMC has received consultancy fees from Puma Biotechnology Inc. JC has been on advisory boards for Puma Biotechnology Inc, Seattle Genetics, and Novartis Ireland. LDE is an employee and shareholder in Puma Biotechnology Inc. All remaining authors have no conflicts of interest to declare.

## Appendix Supplementary materials

Image, application 1
