# Supplementary material for: Neratinib plus dasatinib is highly synergistic in HER2-positive breast cancer in vitro and in vivo
Source: Transl Oncol. 2024 Aug 26;49:102073. doi: 10.1016/j.tranon.2024.102073 (PMC11396364; doi:10.1016/j.tranon.2024.102073)
Supplement: Supplementary file 1 [file mmc1.pptx]

## Slide 1
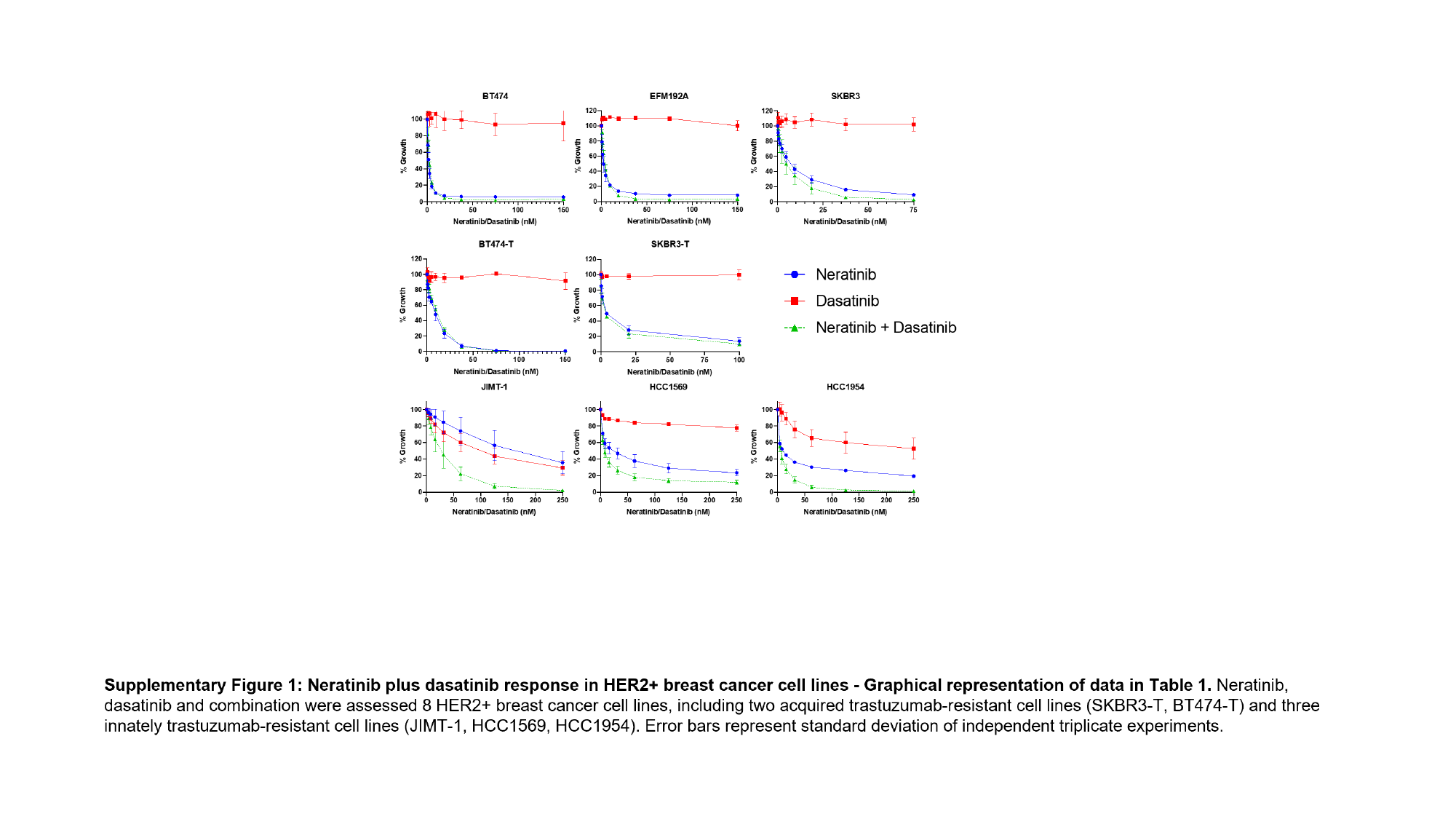

## Slide 2
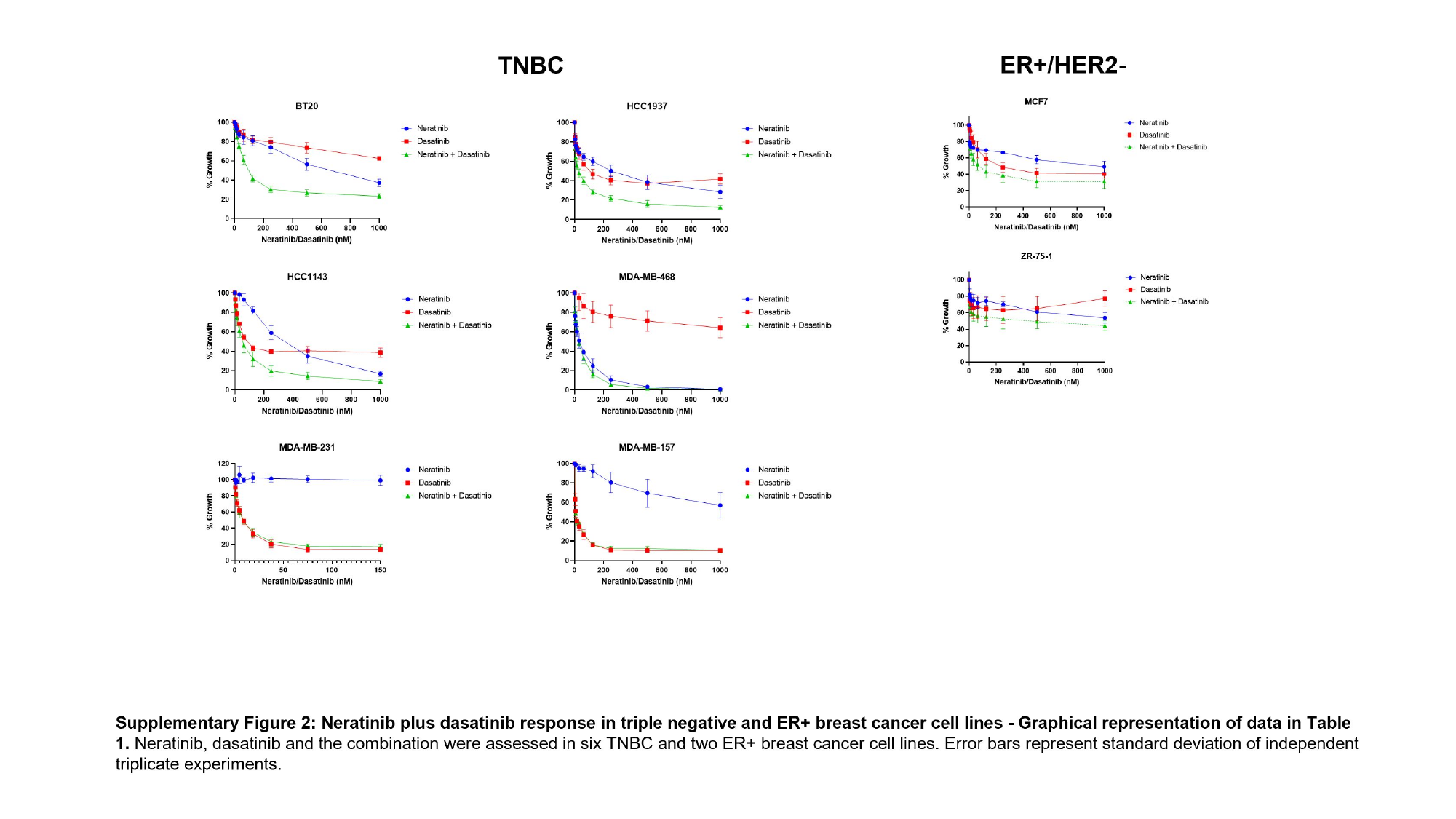

## Slide 3
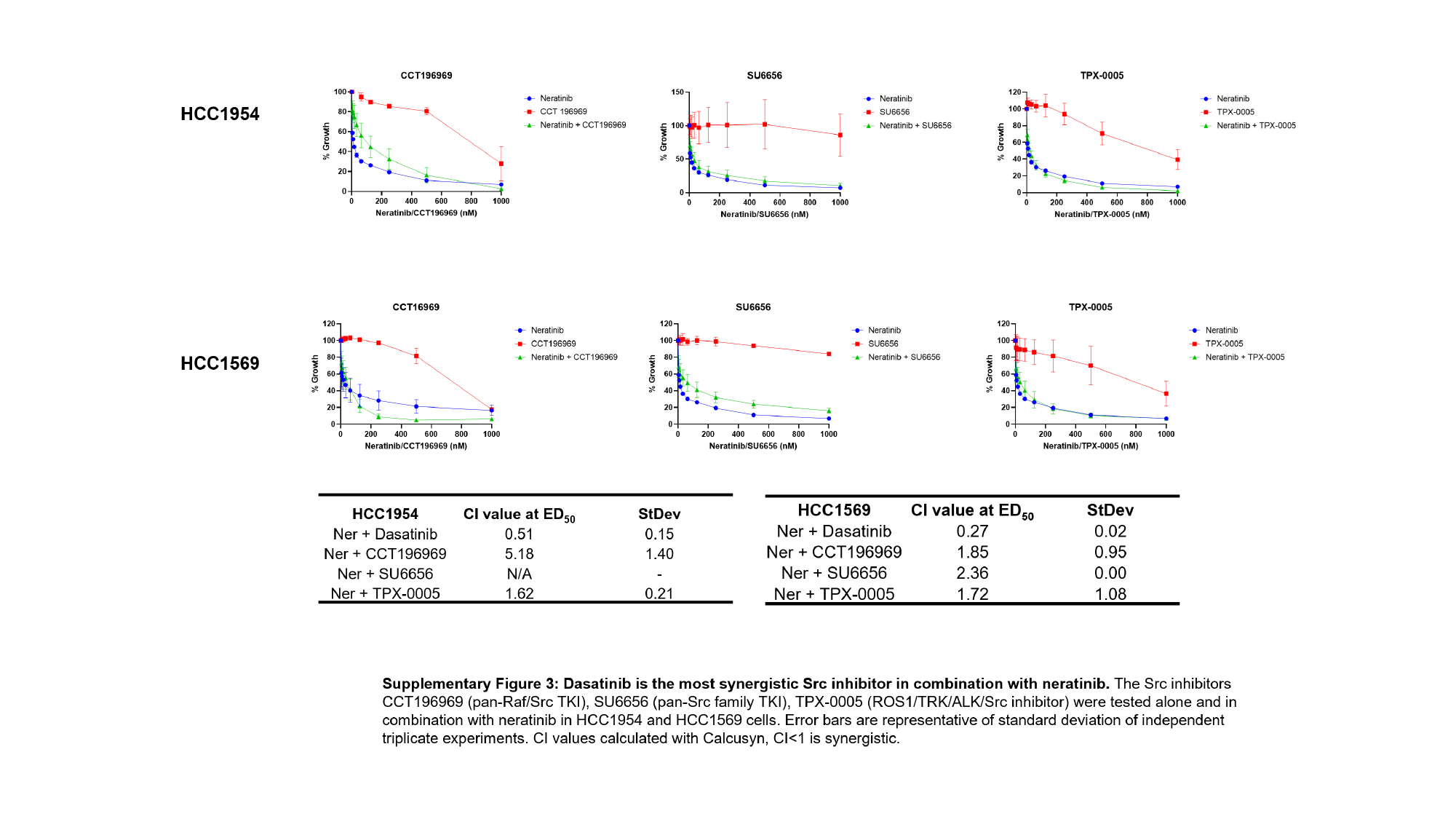

## Slide 4
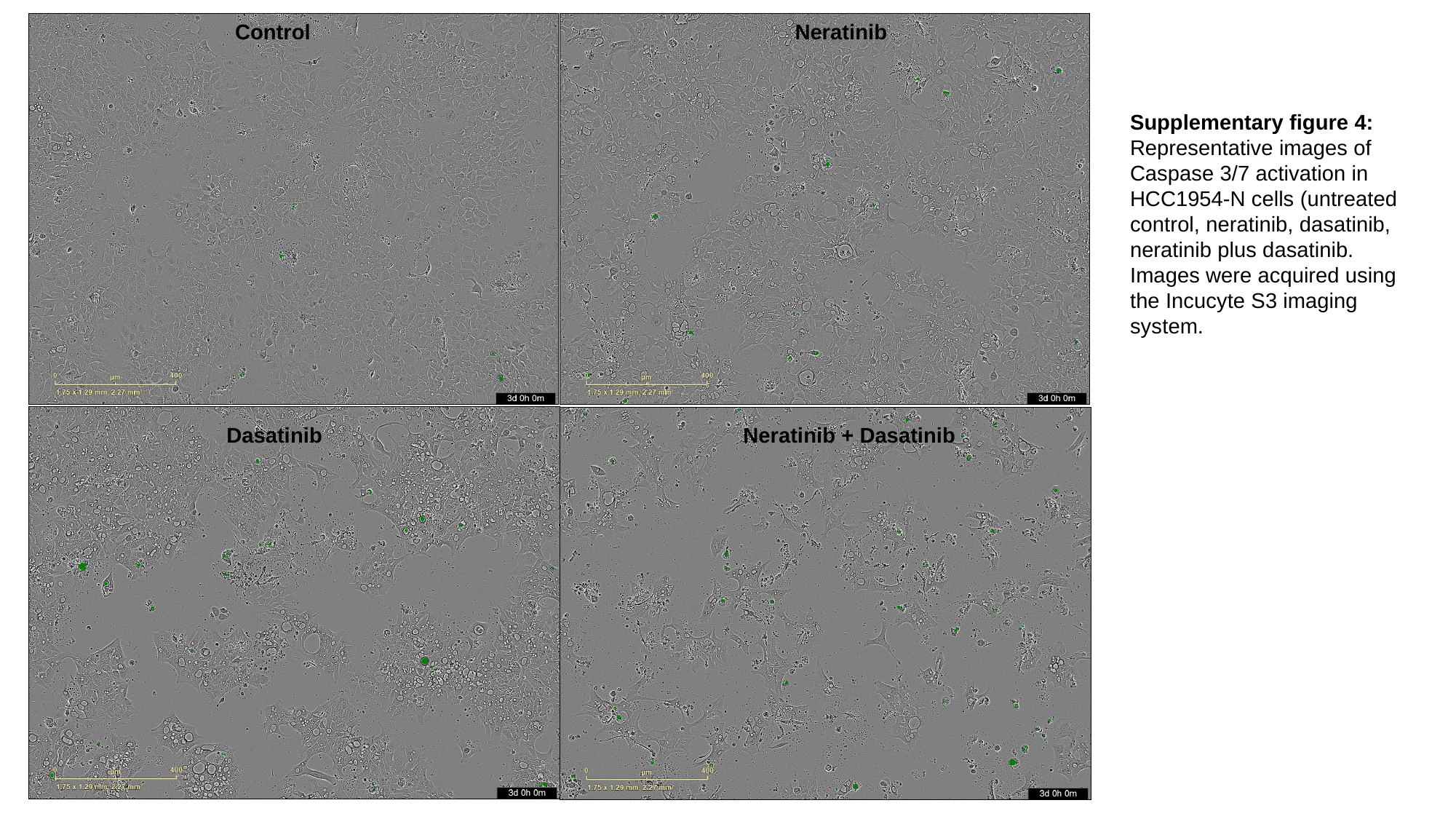

Control
Neratinib
Supplementary figure 4: Representative images of Caspase 3/7 activation in HCC1954-N cells (untreated control, neratinib, dasatinib, neratinib plus dasatinib. Images were acquired using the Incucyte S3 imaging system.
Dasatinib
Neratinib + Dasatinib

## Slide 5
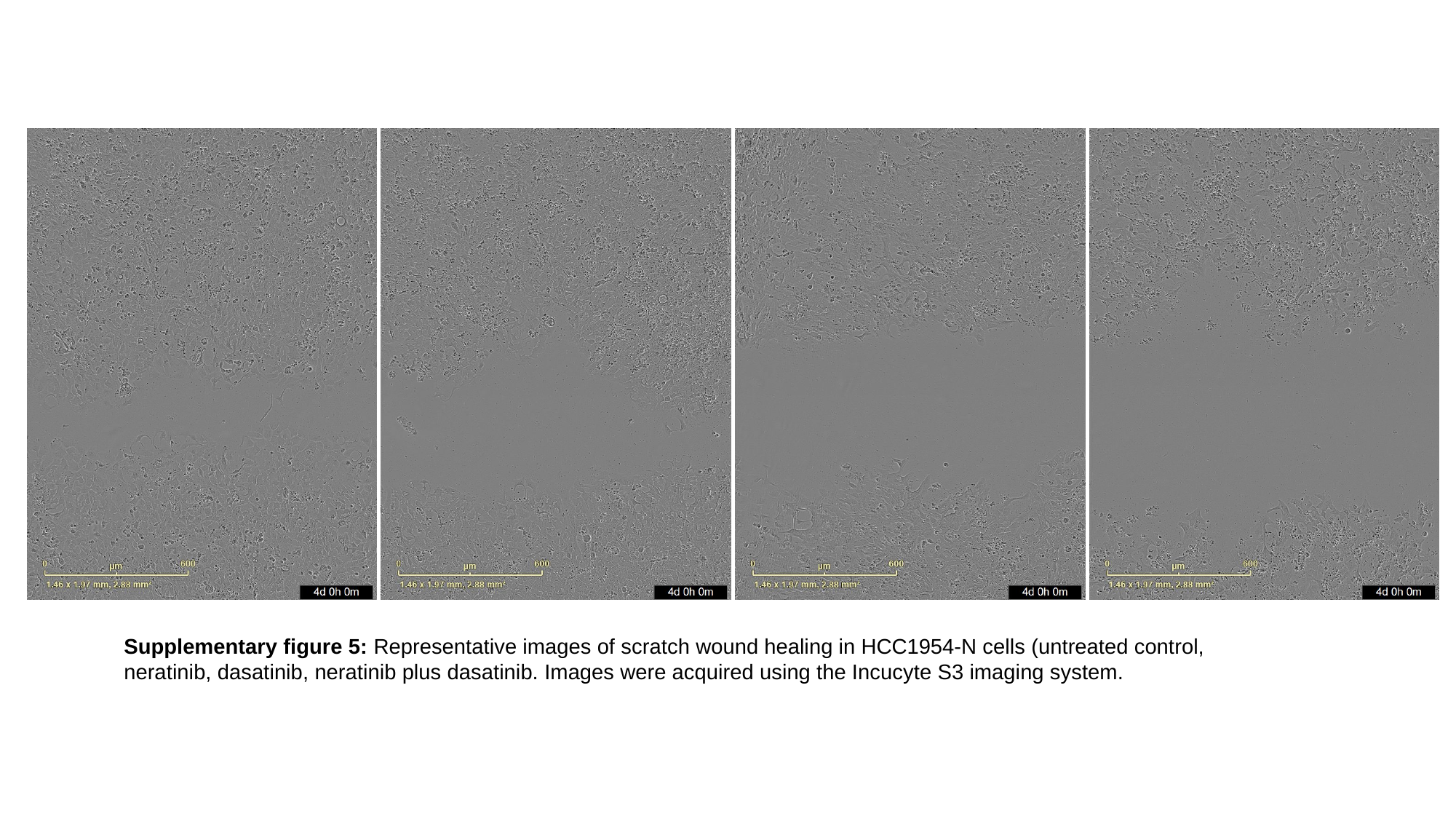

Supplementary figure 5: Representative images of scratch wound healing in HCC1954-N cells (untreated control, neratinib, dasatinib, neratinib plus dasatinib. Images were acquired using the Incucyte S3 imaging system.

## Slide 6
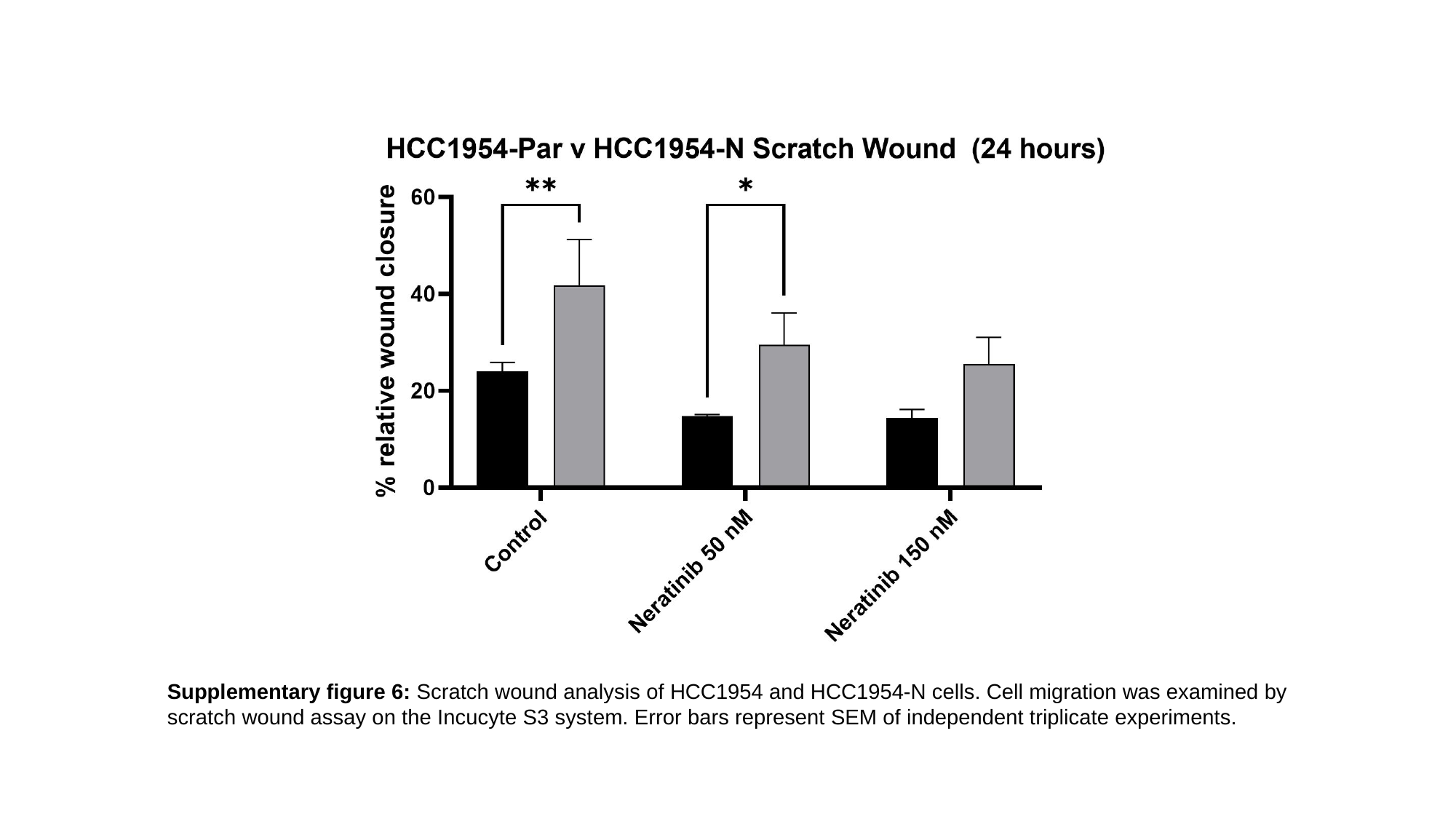

Supplementary figure 6: Scratch wound analysis of HCC1954 and HCC1954-N cells. Cell migration was examined by scratch wound assay on the Incucyte S3 system. Error bars represent SEM of independent triplicate experiments.

## Slide 7
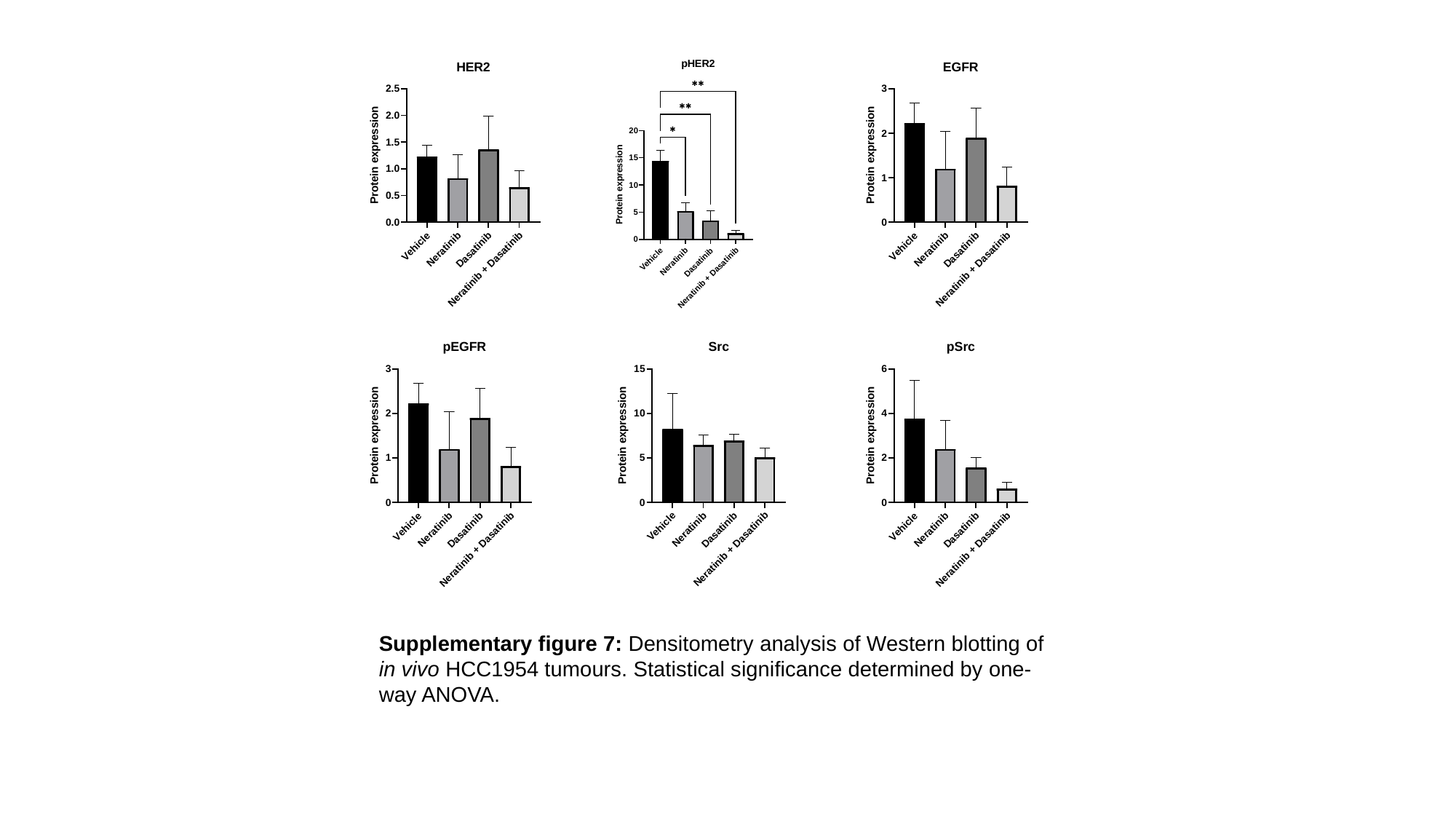

Supplementary figure 7: Densitometry analysis of Western blotting of in vivo HCC1954 tumours. Statistical significance determined by one-way ANOVA.

## Slide 8
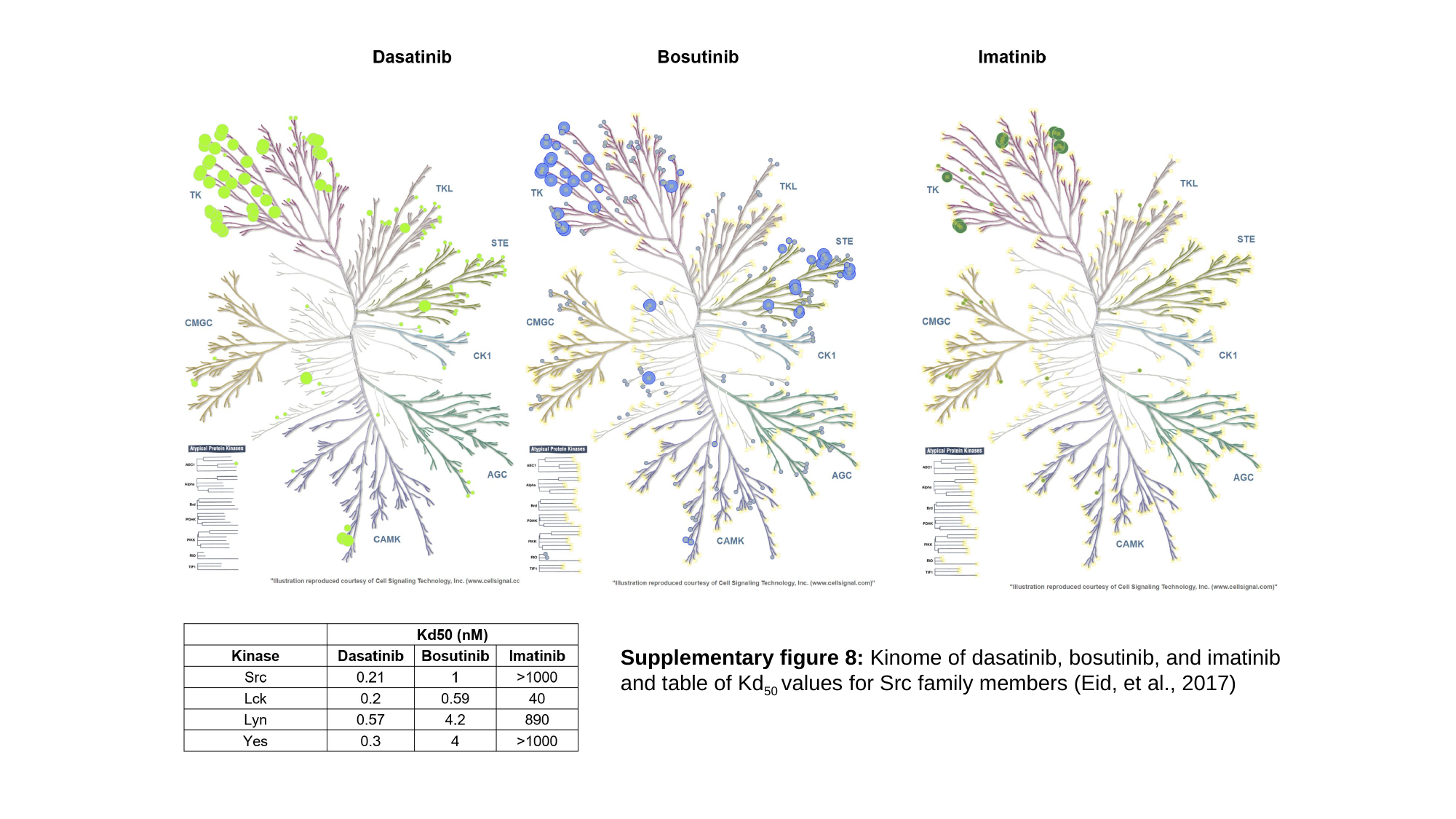

Supplementary figure 8: Kinome of dasatinib, bosutinib, and imatinib and table of Kd50 values for Src family members (Eid, et al., 2017)

## Slide 9
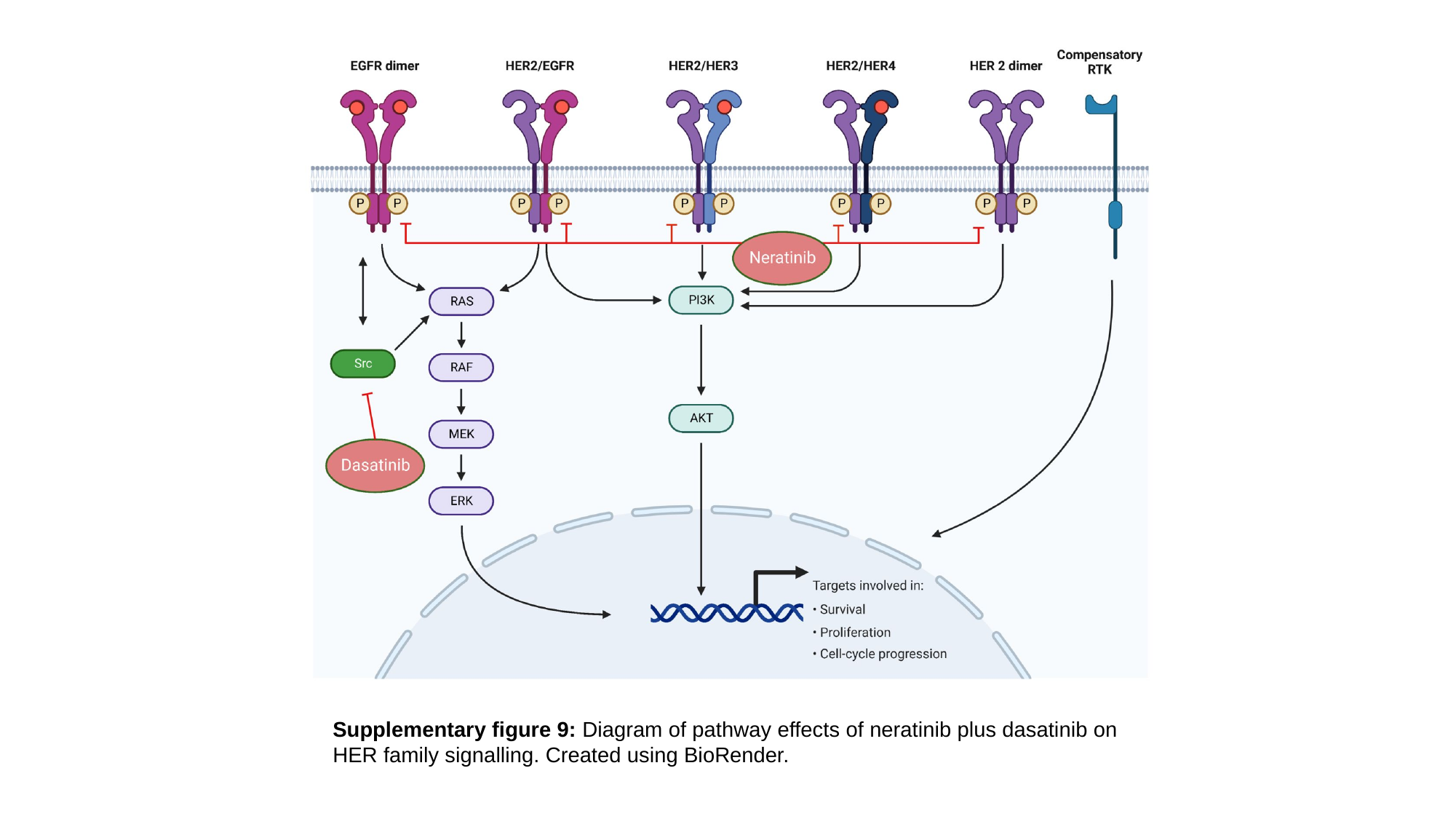

Supplementary figure 9: Diagram of pathway effects of neratinib plus dasatinib on HER family signalling. Created using BioRender.
